# Diagnosis of avulsion fractures of the distal fibula after lateral ankle sprain in children: a diagnostic accuracy study comparing ultrasonography with radiography

**DOI:** 10.1186/s12891-020-03287-1

**Published:** 2020-04-28

**Authors:** Yoshiyuki Takakura, Satoshi Yamaguchi, Ryuichiro Akagi, Makoto Kamegaya, Seiji Kimura, Hirofumi Tanaka, Tetsuro Yasui

**Affiliations:** 1 Takakura Orthopaedic & Sports Clinic, 5-4-21 Tokui-cho, Nada-ku, Kobe-shi, Hyogo 657-0033 Japan; 2 Collage of Liberal Arts and Sciences, Chiba University, 1-8-1 Inohana, Chuo-ku, Chiba-shi, Chiba, 260-8670 Japan; 3 Department of Orthopaedic Surgery, Graduate School of Medical and Pharmaceutical Sciences, Chiba University, 1-8-1 Inohana, Chuo-ku, Chiba-shi, Chiba, 260-8670 Japan; 4 Chiba C & A Orthopaedic Clinic, 3-24-2 Oyumino-minami, Midori-ku, Chiba-shi, Chiba, 266-0033 Japan; 5 Hyakutake Orthopedic Surgery and Sports Clinic, 4-2-15 Mizugae, Saga-shi, Saga, 840-0054 Japan; 6 Department of Orthopaedic Surgery, Teikyo University Mizonokuchi Hospital, 5-1-1 Futako, Takatsu-ku, Kawasaki-shi, Kanagawa 213-8507 Japan

**Keywords:** Lateral ankle sprain, Avulsion fracture, Diagnostic accuracy, Pediatrics, Radiography, Subfibular ossicle, Ultrasonography

## Abstract

**Background:**

The purpose of this study was to determine the diagnostic accuracy of ultrasonography for the diagnosis of avulsion fractures of the distal fibula for lateral ankle sprain in children and compare it to that of radiography.

**Methods:**

Children who sustained lateral ankle sprain were prospectively surveyed. They underwent both ultrasonography and radiography at the first clinic visit to diagnose any concomitant avulsion fractures of the distal fibula. The patients underwent follow-up radiography 4 weeks later to obtain the reference standard diagnosis. The measures of diagnostic accuracy (i.e., sensitivity, specificity, positive predictive value, and negative predictive value) of the initial ultrasonography and radiography were calculated; they were then compared using the McNemar test. Totally, 52 patients (with a median age of 9 years) were analyzed.

**Results:**

On the reference standard (follow-up) radiographs, 32 patients (62%) were found to have avulsion fractures of the distal fibula. The sensitivity, specificity, positive predictive value, and negative predictive value for ultrasonography were 94, 85, 91, and 89% respectively; and 81, 100, 100, and 77% respectively for radiography at the first visit. There were no significant differences in sensitivity and specificity between the two diagnostic methods (*P* = 0.22, 0.25).

**Conclusions:**

Ultrasonography has a high diagnostic accuracy, which is comparable to that of radiography, for the diagnosis of avulsion fracture of the distal fibula. Ultrasonography may be used as an option of imaging modality for lateral ankle sprain in children.

## Background

Lateral ankle sprain is one of the most common injuries that occurs in daily living and sports activities. The incidence rate is higher in children (aged ≤12 years, 2.85/1000 exposures) than in adolescents (aged ≥13 to ≤17 years, 1.94/1000 exposures) and adults (aged ≥18 years, 0.72/1000 exposures) [1]. Avulsion fractures of the distal fibula, which is the insertion of the anterior talofibular ligament (ATFL) and calcaneofibular ligament (CFL) [2], frequently occur concomitantly with lateral ankle sprain in children with an incidence as high as 60 to 70% [3–5]. Several studies have shown that a majority of children with lateral ankle sprain returned to daily activity within 1 to 3 months regardless of the presence of avulsion fracture [6]. However, patients with avulsion fractures have a higher risk of recurrent sprain occurring within 2 years than patients without avulsion fractures [5]. Moreover, most fractures fail to unite and persist as subfibular ossicles [5], which may cause chronic ankle pain and instability, and result in ankle osteoarthritis in the long term [7]. The presence of avulsion fractures could also affect the treatment plan. For ankle sprains without fractures, symptomatic or functional treatment using a brace is appropriate. However, for injuries with avulsion fractures, cast immobilization may be necessary to achieve bone union [8]. Therefore, the presence or absence of avulsion fracture is clinically important, as it can affect the prognosis and treatment of the injury.

Radiography is currently the gold standard for the diagnosis of fractures. However, diagnosis of avulsion fracture is difficult using standard radiographs of the ankle as more than half of the fractures are not visible on anteroposterior and lateral views [5, 9]. Specialized radiographic projections, such as the ATFL and CFL views [10], can visualize avulsion fractures effectively, but at the cost of additional expense and radiation exposure. Although radiography uses low-dose radiation, exposure to this hazard may have a cumulative effect on pediatric patients because the growing bone is vulnerable to radiation injury [11]. Furthermore, small fracture fragments and chondral avulsions may not be detected even if these projections are used [8, 12]. The Ottawa Ankle Rules are commonly used clinical examination rules to identify patients with ankle injuries that do not require radiographic examination [13]. Although the rules have high diagnostic accuracy in excluding ankle fractures, their sensitivity is lower in children than in adults [13]. Therefore, a non-invasive and high accuracy diagnostic tool is needed for the diagnosis of avulsion fracture in children.

With recent improvements in image quality, ultrasonography has been used as a first-line imaging modality for pediatric foot and ankle injuries [14–16]. Sonographic examination of ankle trauma can be immediately performed in an outpatient clinic and reduces the need for radiography [14]. Furthermore, ultrasonography can also visualize small avulsion fractures and unossified epiphyses [8, 14]. Thus, ultrasonography can often reveal a fracture that may overlooked on standard radiographs [17]. These characteristics make it particularly suitable for the diagnosis of avulsion fractures of the distal fibula. Additionally, ultrasonography can visualize ligament injuries that cannot be evaluated by radiographs [18]. However, the diagnostic accuracy of ultrasonography in detecting avulsion fractures of the distal fibula has not been clarified or compared with that of radiography.

The purpose of this study was to determine the diagnostic accuracy of ultrasonography for the diagnosis of avulsion fractures of the distal fibula concomitant with lateral ankle sprain in children and compare to radiography. We hypothesized that the diagnostic accuracy of ultrasonography was comparable to that of radiography.

## Methods

### Patient recruitment

**Fig. 1:**
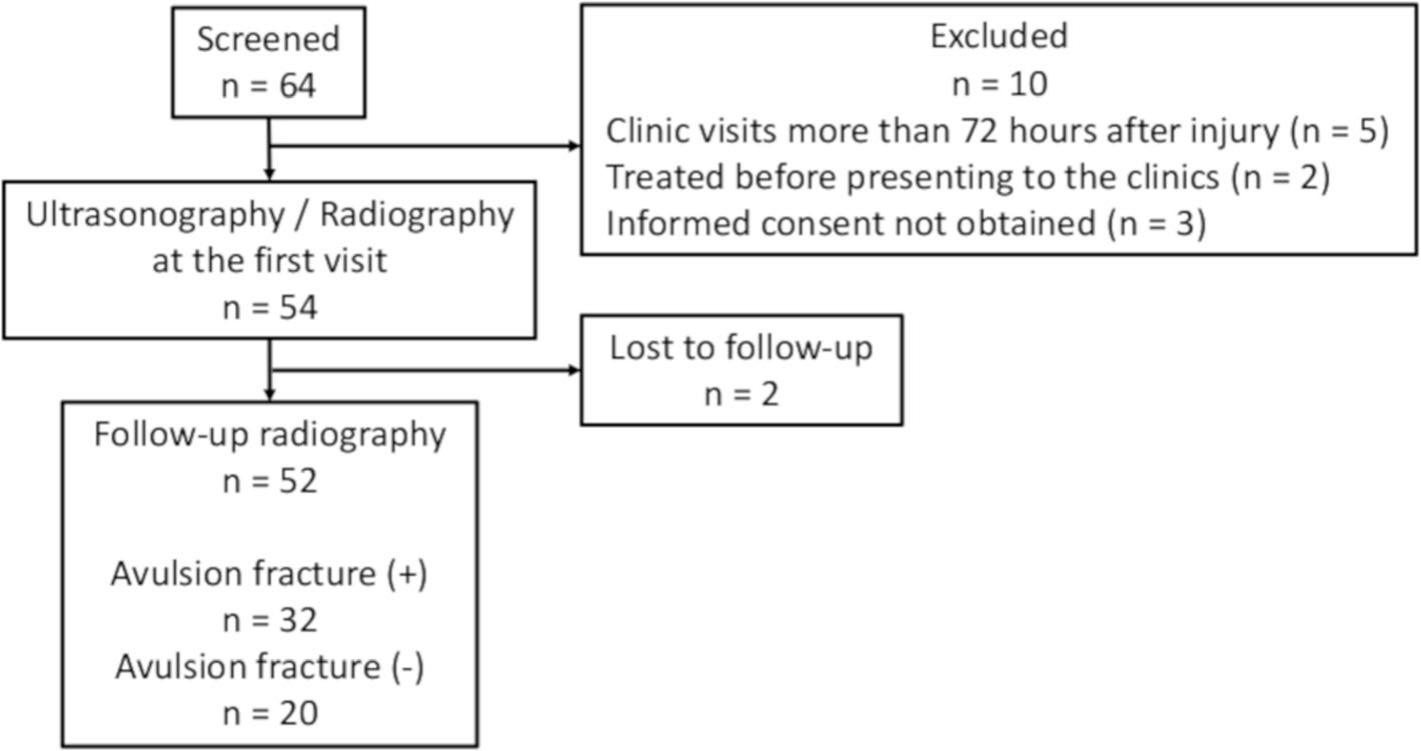
Flow diagram of patients

Patients who visited 4 local orthopaedic clinics complaining of lateral ankle sprain between December 2016 and November 2018 were prospectively screened for eligibility. Inclusion criteria were age between 6 to 12 years [1], inversion ankle sprain, presence of swelling and tenderness localized to the lateral malleolus [19], and inability to walk for 4 or more steps on presentation to the clinic [20]. Exclusion criteria were open fracture, multiple trauma, midfoot injury, history of ankle surgery, and epiphyseal arrest. Patients who visited the clinics more than 72 h after the injury [21, 22] and those who had undergone treatment at other facilities before presenting to the clinics were also excluded. Patients with a history of ankle sprain were not excluded to replicate the clinical setting. Finally, patients who did not consent to participate in this study were excluded. Of the 64 patients screened for eligibility, 10 patients were excluded (Fig. 1). The remaining 54 patients underwent both sonographic and radiographic examinations on the first visit. Two patients were lost to follow-up, and the remaining 52 underwent follow-up radiographs 4 weeks later. There were 28 females and 24 males with a median age of 9 (25th, 75th percentile values; 8, 10) years. Median height, weight, and body mass index were 1.33 (1.25, 1.44) m, 28.0 (24.0, 36.0) kg, and 16.4 (1.54, 17.6) kg/m^2,^ respectively. Eight patients (15%) had a previous history of ipsilateral ankle sprain. The institutional review board of our clinics approved this study, and all subjects and their parents provided written informed consent.

### Ultrasonography

**Fig. 2:**
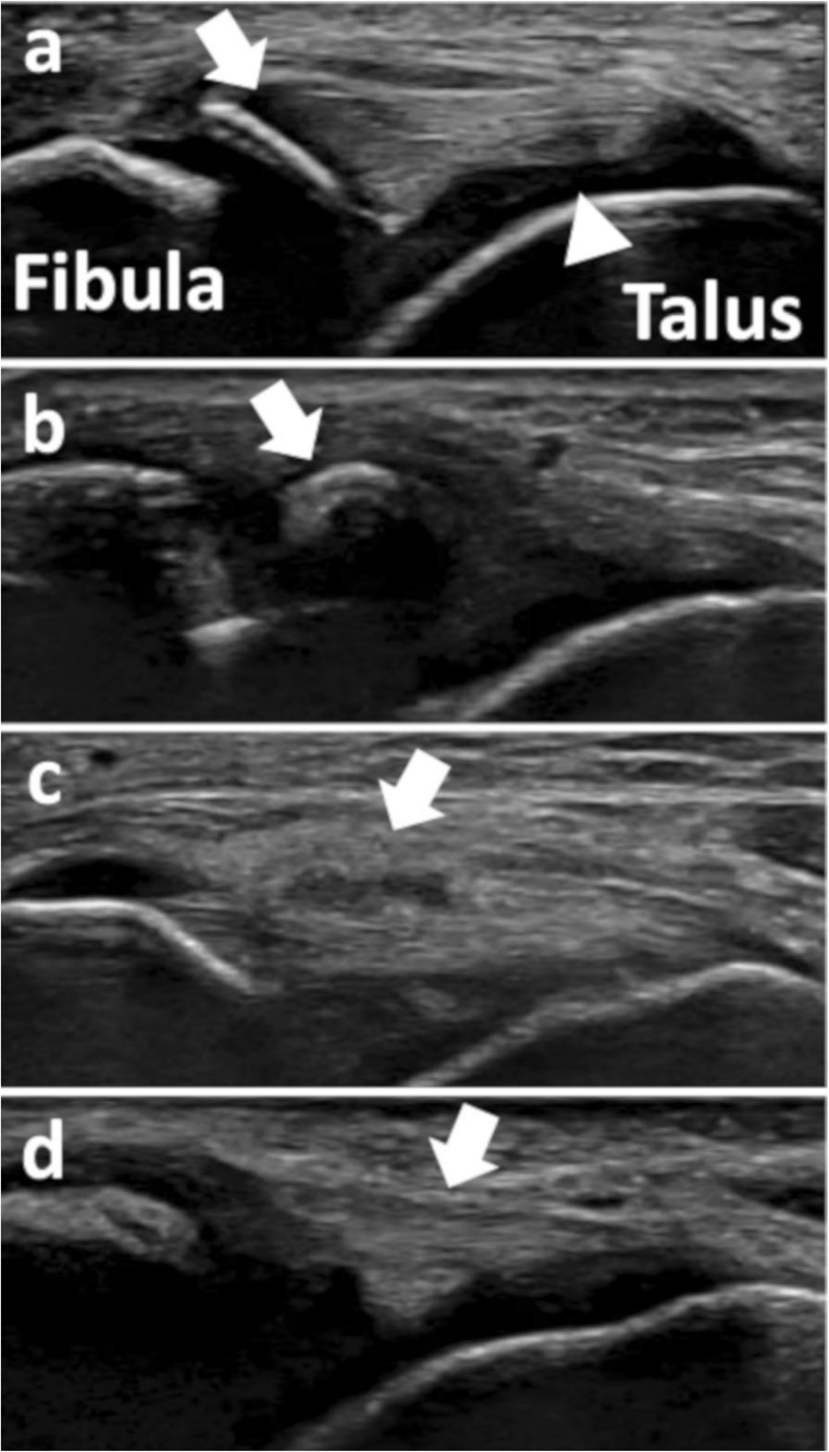
Sonographic image of the longitudinal view of the anterior talofibular ligament. **a** Avulsion fracture (arrow) at the fibular insertion of the anterior talofibular ligament (arrowhead), **b** Subfibular ossicle (arrow), **c** Anterior tibiofibular ligament injury (arrow), **d** No osseous or ligamentous injury (arrow)

Patients underwent ultrasonography at the first visit to clinics, before undergoing radiography. Patients were seated on the edge of the examination bed with the ankle slightly plantarflexed, inverted, and placed on the examiner’s thigh [23, 24]. The probe was placed parallel to the sole to visualize the longitudinal view of the ATFL [18, 23, 24]. Then, the probe was moved proximally and distally to check for avulsion fracture and ATFL injury along the entire width of the ligament [8]. The presence of fractures in other areas of the fibula, defined as disruption or stepping of the cortical bone, was also assessed using axial and longitudinal views of the bone [14]. The examiners scanned both ankles in all subjects because epiphyseal bones with irregular contours often require comparison with the uninjured side for accurate diagnosis [17]. The examination was performed using B mode images. Doppler ultrasonography was not used. The sonographic findings were classified as avulsion fracture of the distal fibula (Fig. 2a), subfibular ossicle (Fig. 2b), epiphyseal injury of the distal fibula, ATFL injury (Fig. 2c), and no ligamentous or osseous injury (Fig. 2d). Avulsion fracture of the distal fibula, as well as epiphyseal fracture, was defined as disruption or stepping of the cortical bone [8, 25]. Subfibular ossicle could include old, nonunited avulsion fractures and secondary ossification centers [26]. While avulsion fractures generally have a shell-like appearance, subfibular ossicles have a round shape with a cortical margin [3]. ATFL injury was defined as a disruption of the fibrillar pattern of the ligament [18]. The diagnoses were further dichotomized into presence or absence of avulsion fracture. Four certified orthopaedic surgeons performed the ultrasonography. Each surgeon had previous experience of more than 3 years with musculoskeletal ultrasonography. The ultrasound machine (LOGIQ e Premium, GE Healthcare, Chicago, Illinois, USA; SONIMAGE HS1, Konica Minolta Healthcare, Marunouchi, Tokyo, Japan) and probe (Linear probe L8-18i-RS, frequency 7–18 MHz, length 35 mm, GE Healthcare, Chicago, Illinois, USA; Linear probe L18–4, center frequency 10 MHz, length 40 mm, Konica Minolta Healthcare, Marunouchi, Tokyo, Japan) varied depending on the clinic. As the 4 examiners were also the treating surgeons, they were not blinded to the subject characteristics and physical examination finding; however, they were blinded to the alternative radiographic findings because the radiographic examination was performed after the ultrasonography. This study setting is representative of how ultrasonography is used in clinical practice.

### Radiography

**Fig. 3:**
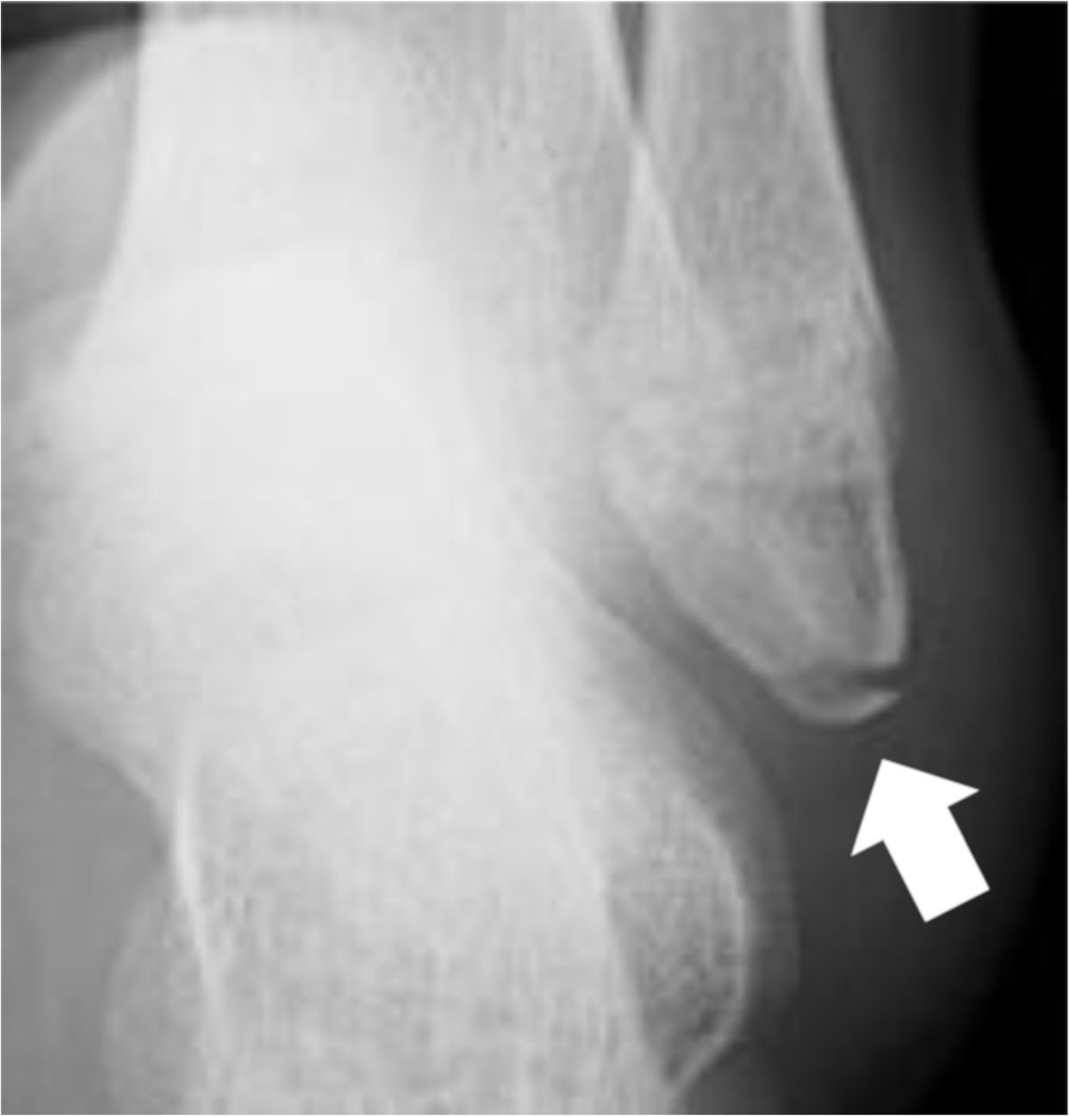
Anterior talofibular ligament view of the ankle. An avulsion fracture of the distal fibula is visualized (arrow)

For all patients, radiographic imaging was performed for both ankles (in mortise, lateral, and ATFL views) on their first visit to the clinics, after ultrasonography. The ATFL view [10] has higher sensitivity for detecting avulsion fractures of the distal fibula than the more conventional mortise and lateral views [5, 9]. The radiographic findings were categorized into avulsion fracture of the distal fibula (Fig. 3), subfibular ossicle, epiphyseal fracture, and no fracture. Diagnosis of avulsion fracture was made if cortical disruption with a shell-like bone fragment was present in at least 1 of the 3 images taken [10]. The diagnoses were further classified according to the presence or absence of avulsion fracture. As the treating surgeons performed the sonographic examination before radiography, they were not able to assess the radiography in a blinded fashion. Therefore, another certified orthopaedic surgeon—who was not involved in the treatment of subjects and was blinded to the sonographic findings—evaluated all radiographic images. The surgeon had an experience of 7 years in assessing musculoskeletal radiographs.

### Reference standard

At least 4 weeks after the first visit, the patients underwent the same set of radiographic examinations on the injured ankle. The treatment method between visits was not standardized and was selected by the patient, their parents, and the treating physician regardless of the presence of avulsion fracture. Treatments ranged from symptomatic treatment with an elastic bandage to the use of a non-weight-bearing below-knee cast for 4 weeks. The evaluation of the follow-up radiographs served as the reference standard for the diagnosis of avulsion fracture. This was because small avulsion fragments, which had not been visible on the initial sonographic and radiographic images, would later ossify and enlarge enough to appear on the follow-up radiographs [12, 27, 28]. Therefore, the follow-up radiographs have been used as the reference standard for the assessment of diagnostic accuracy of ultrasonography for detecting occult ankle fractures [27, 28]. A certified orthopaedic surgeon, who was not involved in the treatment of patients and was blinded to the initial sonographic and radiographic diagnoses, evaluated all radiographic images using the same criteria as that of the initial radiographs. The surgeon had an experience of 17 years in assessing musculoskeletal radiographs. Additionally, we diagnosed the case as fracture if callus formation was noted [26]. The kappa values for intra- and inter-observer reliability in the diagnosis of avulsion fracture were 1.00 and 0.92, respectively.

### Statistics

Sensitivity, specificity, positive predictive value (PPV), and negative predictive value (NPV) (with 95% confidence interval) for the diagnosis of avulsion fracture of the distal fibula were calculated for ultrasonography and radiography at the first visit, by comparison to the follow-up radiography (i.e. the reference standard). The diagnostic performance measures were calculated using the following equations:

**Table:** 

Ultrasonography/radiography at first visit	Radiography at follow-up (Reference standard)	
	Avulsion fracture (+)	Avulsion fracture (−)
Avulsion fracture (+)	True-positive (TP)	False-positive (FP)
Avulsion fracture (−)	False-negative (FN)	True-negative (TN)

Sensitivity = TP/(TP + FN)

Specificity = TN/(TN + FP)

PPV = TP/(TP + FP)

NPV = TN/(FN + TN)

The sensitivities and specificities of the diagnoses made using ultrasonography and radiography were compared using a two-sided McNemar test [29]. Agreement between the diagnoses made using ultrasonography and radiography done at the first visit was assessed using kappa statistics. Furthermore, the diagnostic performance measures for the overall diagnoses, including those of subfibular ossicle and epiphyseal fracture were calculated. A *P* value of less than 0.05 was considered statistically significant.

## Results

On the follow-up (reference standard) radiographs, 32 patients (62%) were diagnosed as avulsion fractures of the distal fibula (Table 1). On the ultrasonography and radiography at the first visit, 33 (63%) and 26 (50%) patients were diagnosed with avulsion fractures, respectively.

**Table 1:** Diagnosis of ultrasonography and radiography at the first visit and follow-up (*n* = 52)

	Ultrasonography at first visit	Radiography at first visit	Radiography at follow-up (Reference standard)
Avulsion fracture at the distal fibula	33 (63)	26 (50)	32 (62)
Subfibular ossicle	2 (4)	2 (4)	1 (2)
Epiphyseal fracture	2 (4)	0 (0)	0 (0)
Anterior talofibular ligament injury	5 (10)	n.a.	n.a.
No osseous (or ligamentous) injury	10 (19)	24 (46)	19 (37)

Values show the number (percent) of patients

*n.a.* not applicable

The sensitivity and specificity of ultrasonography were 94% (95% confidence interval; 79, 99%) and 85% (62, 97%) respectively, with the PPV and NPV at 91% (76, 98%) and 89% (67, 99%) respectively (Table 2). Of the 2 false-negative diagnoses, 1 was diagnosed as subfibular ossicle and the other 1 was diagnosed as no injury on the initial ultrasonography. For the 3 false-positive diagnoses, the diagnoses of the follow-up radiographs were no injury in all cases. The sensitivity and specificity of radiography were 81% (64, 93%) and 100%, respectively, with the PPV and NPV of 100 and 77% (56, 91%), respectively (Table 3). Of the 6 false-negative diagnoses, 1 was diagnosed as subfibular ossicle and 5 were diagnosed as no injury on the initial radiographs. There were no statistical differences in the sensitivities and specificities between the two diagnostic imaging techniques (*P* = 0.22 and 0.25 for sensitivity and specificity, respectively). The kappa coefficient for the agreement of ultrasonographic and radiographic diagnoses at the first visit was 0.65 (0.46, 0.85).

**Table 2:** Diagnosis of avulsion fracture using ultrasonography at the first visit in relation to reference diagnosis using follow-up radiography (*n* = 52)

Ultrasonography at first visit	Radiography at follow-up (Reference standard)		Total
	Fracture (+)	Fracture (−)	
Avulsion fracture (+)	30	3	33
Avulsion fracture (−)	2	17	19
Total	32	20	

Values show the number of patients

**Table 3:** Diagnosis of avulsion fracture using radiography at the first visit in relation to reference diagnosis using follow-up radiography (*n* = 52)

Radiography at first visit	Radiography at follow-up (Reference standard)		Total
	Fracture (+)	Fracture (−)	
Avulsion fracture (+)	26	0	26
Avulsion fracture (−)	6	20	26
Total	32	20	

Values show the number of patients

Of the 26 patients who were diagnosed with avulsion fracture of the distal fibula on the initial radiographs at the initial visit, all fractures were visible on the ATFL view. However, only 15 fractures were depicted on the standard mortise and lateral views.

The sensitivity and specificity of ultrasonography for overall diagnosis, including those of subfibular ossicle and epiphyseal fracture, were 94% (79, 99%) and 75% (51, 91%), respectively, with the PPV and NPV of 86% (70, 95%) and 88% (64, 99%) (Table 4). In addition to the 3 false-positive diagnoses for avulsion fracture as described above, the ultrasonographic diagnosis of epiphyseal fracture was determined to be a false positive due to the absence of callus or periosteal reaction on the follow-up radiographs. The diagnostic performance measures of radiography for overall diagnosis were the same as those for the diagnosis of avulsion fracture.

**Table 4:** Diagnosis of overall fracture using ultrasonography in relation to reference diagnosis using follow-up radiography (*n* = 52)

Ultrasonography at first visit	Radiography at follow-up (Reference standard)		Total
	Fracture (+)	Fracture (−)	
Overall fracture (+)	30	5	35
Overall fracture (−)	2	15	17
Total	32	20	

Values show the number of patients

## Discussion

We showed that the diagnostic accuracy of ultrasonography for the diagnosis of avulsion fracture of the distal fibula was comparable to that of radiography, with a sensitivity and specificity of over 85%. Ultrasonography may be used as the first-line imaging investigation for lateral ankle sprain in children as an alternative to radiography.

The sensitivity and specificity of ultrasonography was 94 and 85%, respectively. These values were comparable to those of sonographic diagnosis of foot and ankle fractures in adults [15, 30, 31], although, in these studies, most fractures were malleolar and metatarsal fractures. Szczepaniak et al. [25] performed ultrasonography on 212 children who had sustained lateral ankle sprains. They detected avulsion fractures in 29% of patients; however, the authors [25] did not report the diagnostic accuracy of ultrasonography. Najaf-Zadeh et al. [27] reported in their meta-analysis on occult fractures in children, that the sensitivity and specificity of ultrasonography for the diagnosis of occult ankle fractures were 100 and 95% respectively. Unfortunately, avulsion fractures of the distal fibula were not assessed. Our study showed that ultrasonography was also effective in detecting avulsion fractures.

Although the overall diagnostic accuracy of ultrasonography was as high as that of radiography, there were 3 false-positive and 2 false-negative results in the diagnosis of avulsion fracture. Additional false-positive diagnoses, in which sonographic diagnosis of epiphyseal fracture of the lateral malleolus was determined to be no osseous injury according to the follow-up radiographs, occurred in 2 patients. Both false-positive and false- negative results have been reported in the sonographic diagnosis of foot and ankle fractures in adults [15, 16, 30]. The use of Doppler sonography could improve the accuracy because Doppler signals appear around the acute fracture site due to hematoma and increased blood flow [4, 32]. Therefore, it may be used to distinguish an acute avulsion fracture from a subfibular ossicle, and to distinguish an epiphyseal fracture from a natural irregularity of epiphysis; however, further research is necessary to clarify the usefulness of Doppler ultrasonography [32]. Furthermore, treating physicians should inform patients and their parents about the possibility of a false positive or false negative diagnosis.

Even though the ATFL views were obtained in this study, the sensitivity of radiography for the diagnosis of avulsion fracture was 81%: 6 of the 26 avulsion fractures were missed on the initial radiographs. One possible reason for the relatively high incidence of false negative diagnoses was that chondral avulsions were not visualized on the initial radiographs. These later ossified and appeared on the follow-up radiographs [12, 27, 28]. Although controversy exists, accurate diagnosis of avulsion fractures of the distal fibula would be clinically important because Yamaguchi et al. [5] showed that the incidence rate of recurrent sprain was 44% in patients with avulsion fractures, as compared to 23% in those without fractures. Vahvanen et al. [33] also reported the poorer clinical outcome in patients with avulsion fractures than in those without fractures. Therefore, patients and their parents should be informed that the avulsion fracture may become evident on follow-up radiographs even though the fracture was not clear on the initial radiographs.

There were no significant differences in sensitivity and specificity between the two diagnostic imaging techniques. Thus, treating physicians can select either modality for the diagnosis of avulsion fracture, based on their experience and the availability of the equipment. Ultrasonography has several advantages over radiography [17]; ultrasonography does not expose the patient to radiation. It is immediately available in the outpatient room and can be used even outside the clinics with portable machines. It also leads to substantial cost reduction when used appropriately. Additionally, soft tissue injuries associated with bone injury can be assessed simultaneously. However, it is not clear if ultrasonography can also be used for the follow-up examination such as for the assessment of fracture healing. Therefore, ultrasonography cannot fully replace radiography but it may be used as a screening tool to decrease the risk of radiation hazard [14].

With the recent development of high-frequency ultrasonography transducers, the role of ultrasonography in orthopaedic surgery, not only as a diagnostic tool but also as a therapeutic and research tool, has been increasingly recognized [34]. Similar to other medical fields [35], surgeon-performed ultrasonography has become increasingly common among orthopaedic surgeons. However, we acknowledge that it would be difficult for many orthopaedic surgeons to perform ultrasonography on a routine basis due to limited time, cost, and experience. In these circumstances, radiologists and other experienced examiners can perform ultrasonography [36]. Further research is needed on the clinical availability of ultrasonography, although this issue is beyond the scope of this study.

This study has several limitations. Firstly, the certified orthopaedic surgeons, with experiences of more than 3 years in musculoskeletal ultrasonography, performed the ultrasonography. Therefore, the results of this study may not be applicable if less experienced examiners perform ultrasonography [36]. Secondly, we did not assess the reliability of the sonographic diagnosis over time due to limited time, as the examiners were in a clinical practice setting. Studies have shown that the intra-and inter-rater reliability of foot structure assessment is satisfactory [37, 38]. However, further research is warranted to clarify the reliability of fracture detection, although repeated examinations would be difficult on pediatric trauma patients. Thirdly, 2 patients who underwent the index examinations were lost to follow-up radiography; this might have affected the diagnostic accuracy. Fourthly, the ultrasound machines were different among the clinics, which might have affected the diagnostic performance. Finally, clinical information, such as physical examination findings, was available to the sonographic examiners because they were the treating surgeons.; however, it was not available to the radiographic examiners. Therefore, the diagnostic accuracy of radiography might have been lower than that routinely observed in clinical practice. Finally, the number of patients was relatively small.

## Conclusions

In conclusion, ultrasonography, performed by experienced examiners, has a high diagnostic accuracy, which is comparable to that of radiography for the diagnosis of avulsion fracture of the distal fibula. Ultrasonography may therefore be used as an alternative to radiography for lateral ankle sprain in children.

## Data Availability

The datasets used and/or analyzed during the current study are available from the corresponding author on reasonable request.

## Abbreviations

ATFL: Anterior talofibular ligament
CFL: Calcaneofibular ligament
